# Pathogenetic Role and Clinical Implications of Regulatory RNAs in Biliary Tract Cancer

**DOI:** 10.3390/cancers13010012

**Published:** 2020-12-22

**Authors:** Nduka Ofoeyeno, Emmanuel Ekpenyong, Chiara Braconi

**Affiliations:** 1 The Institute of Cancer Sciences, University of Glasgow, Glasgow G61 1QH, UK; nduka.ofoeyeno@gmail.com; 2 Faculty of Medicine, University of Calabar, Calabar 540271, Nigeria; emmanuelnsa.nsa@gmail.com; 3 Beatson West of Scotland Cancer Centre, Glasgow G12 Y0N, UK

**Keywords:** non-coding RNA, biliary tract cancer, miRNA, biomarker, therapy, LncRNA

## Abstract

**Simple Summary:**

Non-coding ribonucleic acid (RNA) are small molecular structures with relevant functions in various disease processes. They might serve as biomarkers for the diagnosis and as targets for the treatment of biliary tract cancer. This is particularly relevant because current diagnostic methods of the condition only allow for late detection of the disease and therapeutic options are limited. This review aims to explore the possibilities of using non-coding RNA, in particular microRNA, to reduce mortality due to biliary tract cancer through earlier diagnosis of the condition and development of effective therapy.

**Abstract:**

Biliary tract cancer (BTC) is characterised by poor prognosis and low overall survival in patients. This is generally due to minimal understanding of its pathogenesis, late diagnosis and limited therapeutics in preventing or treating BTC patients. Non-coding RNA (ncRNA) are small RNAs (mRNA) that are not translated to proteins. ncRNAs were considered to be of no importance in the genome, but recent studies have shown they play essential roles in biology and oncology such as transcriptional repression and degradation, thus regulating mRNA transcriptomes. This has led to investigations into the role of ncRNAs in the pathogenesis of BTC, and their clinical implications. In this review, the mechanisms of action of ncRNA are discussed and the role of microRNAs in BTC is summarised. The scope of this review will be limited to miRNA as they have been shown to play the most significant roles in BTC progression. There is huge potential in miRNA-based biomarkers and therapeutics in BTC, but more studies, research and technological advancements are required before it can be translated into clinical practice for patients.

## 1. Introduction

The incidence of biliary tract cancer (BTC) is on the rise worldwide [1], likely attributable to the rise in risk factors such as biliary cysts, lithiasis, hepatitis and metabolic syndrome [2]. The incidence rate of BTC varies globally mainly due to the different risk factors across different parts of the world [3,4]. BTC include cholangiocarcinoma (CCA), gall bladder cancer (GBC) and ampullary cancers (AC). According to recent evidence, the increase in incidence seems to be related mainly to an increased prevalence of intrahepatic CCA over the last decades [5]. The clinical prognosis of BTC is poor with GBC patients having the worst survival [6]; overall most patients live less than 18 months despite treatment. The dismal prognosis is due to a limited understanding of the pathogenesis of BTC, late diagnosis of disease and limited therapeutic options. There is, therefore, a pressing need in research to develop novel clinical biomarkers to facilitate the early detection of BTC as well as therapeutics to improve prognosis and overall survival.

## 2. Non-Coding RNA

About 80% of all genes are biologically active in humans and can subsequently be transcribed into messenger RNA (mRNA), but only ~3% of genes transcribed into mRNA can be further translated into proteins [7]. The mRNA transcripts that are translated into protein are referred to as the coding RNAs, while mRNAs that are not translated into protein are referred to as ncRNA [8]. There are mainly two types of ncRNAs, namely long non-coding RNA (lncRNA) and small ncRNA. The latter include microRNA, small interfering RNA (siRNA), piwi RNA small nucleolar RNA (snoRNA), small nuclear RNA (snRNA) [9]. Non-coding RNA (ncRNA) was once considered the “junk” of messenger RNA but has been shown over the last years to have a more important role in biology and oncology than was previously thought. ncRNAs are known to carry out various functions in the cell including transcriptional repression, degradation and regulation of translation of coding RNA [8]. Recent studies have also demonstrated that ncRNA play important roles in pathways leading to pre-cancerous diseases [10,11,12,13,14]. In this manuscript, we will review the role of ncRNA in BTC from a pathogenic point of view and discuss the clinical implications of ncRNA as diagnostic biomarkers as well as targets for therapeutics.

### 2.1. Long Non-Coding RNA

lncRNAs are ncRNAs with a size of about >200 nucleotides and 100 kilobases in length [15]. In the NONCODE database, there are approximately about 10,000 lncRNA genes in the human genome but this is most likely an underestimated figure [16]. lncRNAs have been shown to have an integral role in many biological processes. Examples include epigenetic regulation of gene expression, embryonic development, cancer, cell cycle, cell structure integrity, genomic imprinting, cell differentiation, heat shock process, etc. [17,18,19,20,21,22]. lncRNAs execute their functions in several ways namely acting as molecular signals, decoy RNAs, guide probes, and scaffolds. 

lncRNA can be divided into six different subtypes according to the genomic loci from which the lncRNA is transcribed: intronic lncRNA, sense lncRNA, anti-sense lncRNA, long intergenic lncRNA, bidirectional lncRNA and miscellaneous lncRNA [15]. Transcription of lncRNA genes is carried out by several RNA polymerases including RNA polymerase II, RNA polymerase III and RNA polymerase IV [23,24]. The earliest study to illustrate the epigenetic role of lncRNAs was a study by Brown et al. who investigated the role of XIST lncRNA in X chromosome [25]. XIST is a lncRNA of 17 kb in size and was shown to be involved in X inactivation by binding the X chromosome in the nucleus and subsequently inactivating the gene in cis via coating it [22,25]. Numerous studies since then demonstrated the role of lncRNAs in modulating DNA replication and RNA expression in several physiological processes that cover a range of diseases such as neurodegenerative disease, cancer, Alzheimer’s disease, kidney disease and heart disease [26,27,28,29,30,31,32,33]. 

There is evidence for the diverse roles of lncRNA in BTC, but it is mainly based on preclinical evidence [34,35,36]. For this review, emphasis will be placed on miRNA because there is more well-defined evidence for its role in BTC pathogenesis and clinical usefulness.

### 2.2. microRNA

**Figure 1:**
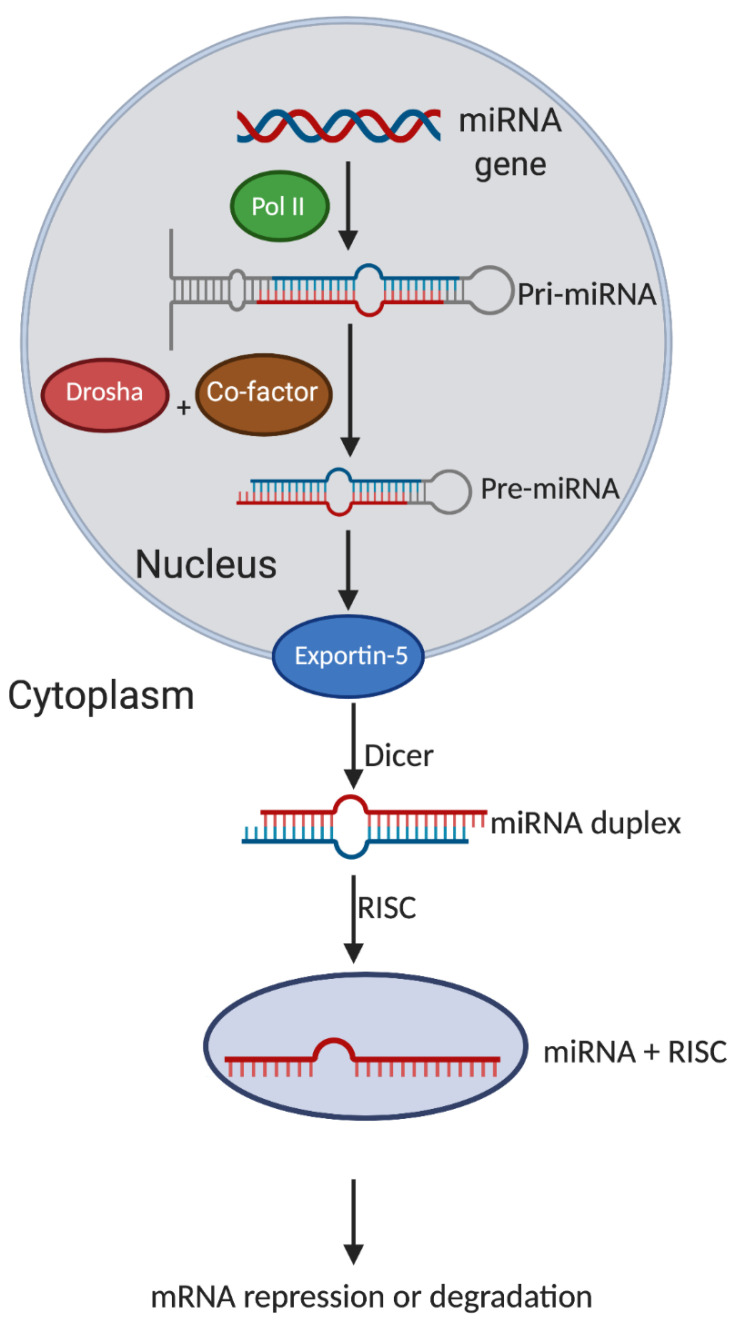
A schematic illustration of miRNA biogenesis and mechanism of action. miRNA gene is transcribed by RNA polymerase II into primary miRNA (pri-miRNA). Pri-miRNAs are then processed in the nucleus by an enzyme named Drosha which has an RNAse III domain and a co-factor into miRNA precursors (pre-miRNAs). The pre-miRNAs are exported from the nucleus to the cytoplasm by Exportin-5 and co-factor RAN-GTP. The hairpin structure of pre-miRNA is cleaved off by Dicer to produce the miRNA duplex. RNA inducing silencing complex (RISC) binds with the long messenger RNA (mRNA) and can subsequently either repress or degrade mRNA.

microRNAs (miRNAs) are endogenous single-stranded ncRNAs that have a size of approximately 18–26 nucleotides in length [37,38]. The synthesis of miRNAs begins with the transcription of primary miRNAs (pri-miRNA) by the RNA polymerase II [39]. Pri-miRNAs contain about 80 nucleotides with a semi-loop hairpin structure and have a 5′CAP structure and poly-adenylated tail [39,40]. Pri-miRNAs are subsequently processed in the nucleus by an enzyme named Drosha which has an RNAse III domain and a co-factor into miRNA precursors (pre-miRNAs) [41,42]. The pre-miRNAs which are about 60–84 nucleotides long are subsequently transported out of the nucleus to the cytoplasm by the nuclear export factor exportin-5 and cofactor RAN-GTP [43,44]. This change in cell localisation of pre-miRNA induces the recruitment of DICER, a type III RNA that processes the pre-miRNA and cleaves off the hairpin structure of the miRNA [45,46]. A protein called argonaute is subsequently recruited to form the RNA inducing silencing complex (RISC) where the miRNA interacts and binds to complementary long mRNA [47,48] (Figure 1).

miRNAs were originally thought to have two mechanisms of action by which they carry out their regulatory function. Firstly, by binding near perfectly to the complementary protein-coding mRNA, the miRNA could induce the RISC to cut and delete the target mRNA [49]. Secondly, by binding imperfectly to the 3′ untranslated region of the protein-coding mRNA, the miRNA could induce translational inhibition and target mRNA degradation [50,51]. Recent studies have now shown miRNAs have a much broader mechanism of action than originally thought. miRNA can bind also to the 5′ untranslated region, inhibiting or promoting the expression of the target mRNA. miRNAs can also induce upregulation of translation by either directly recruiting protein complexes that activate translation or indirectly by inhibiting the translational activity of an inhibitor protein [15,52]. miRNAs have been shown to have an agonistic effect on toll-like receptors and affect toll-like receptor signalling and downstream effect [53]. Current studies also show that miRNAs can be secreted in exosomes or micro-vesicles into bodily fluids, e.g., plasma, serum bile, breast milk, etc. and thus can act as paracrine or endocrine factors [54]. MiRNA are highly conserved across species and rarely found mutated. However, mutations in the main components of the miRNA biogenesis machine, such as DICER, has shown to induce a genome wide deregulation of the microRNome with cancer-specific miRNA signatures associated to given mutational hotspot in DICER [55].

Additionally, miRNAs play significant roles in a variety of physiological conditions and diseases such as inflammation, heart disease, kidney disease, obesity and cancer [56,57,58,59,60,61,62]. 

## 3. Roles of miRNA in Biliary Tract Cancer

miRNA upregulation or downregulation is known to affect BTC in a variety of ways. miRNAs can be grouped into oncogenic miRNAs and tumour suppressor miRNA depending on their targets. The oncogenic miRNAs are upregulated in BTC (e.g., miR-21, miR-25, miR-1249) and can induce a variety of actions that promotes carcinogenesis. These include inducing resistance to apoptosis, sustaining proliferative signalling, inducing invasiveness and metastasis of cancer cells, promoting a tumour microenvironment, etc. Tumour suppressor miRNA, e.g., miR-148, miR-152, miR-494 have been shown to suppress cancer growth in BTC in several ways which include inhibiting cell cycle progression, inducing apoptosis of cancer cells, etc. These tumour suppressor miRNAs are often downregulated or under-expressed in BTC to facilitate cancer growth [63]. Here, some well-defined oncogenic and tumour suppressor miRNAs dysregulated in BTC will be discussed (Table 1 and Table 2).

### 3.1. Oncogenic miRNA

miR-21: foundational evidence of the role miRNA plays in carcinogenesis in CCA was discovered in a study by Meng et al. [64] showing an overexpression of miR-21 in CCA cells in comparison to normal cells. A different study by Selaru et al. confirmed an overexpression of miR-21 in human CCA tissues in relation to non-cancer tissues [65] and showed that miR-21 induced carcinogenesis in CCA by downregulating the expression of programmed cell death 4 (PDCD4) and tissue inhibitor of metalloproteinase (TIMP) 3. Wang et al. showed that miR-21 plays a crucial role in iCCA by upregulating proteins involved in cell cycle and cell proliferation such as protein tyrosine phosphatase (PTEN) and protein tyrosine phosphatase non-receptor type 14 (PTPN14) [66]. The study also illustrated that inhibition of miR-21 expression in iCCA in vitro and in vivo induced cell cycle arrest and apoptosis, while overexpression of miR-21 lead to poor prognosis and adverse clinical outcome such as lower survival rates.

miR-21 has also been shown to induce carcinogenesis by inducing a pro-inflammatory environment in BTC. For example, cyclooxygenase (COX)-2 derived prostaglandin E2 (PGE2) is the most expressed prostaglandin in cancers and is known to be associated with increase cell proliferation and migration. NAD+ linked 15-hydroxy-prostaglandin dehydrogenase (15-PDGH) is an enzyme that acts as a tumour suppressor by converting PGE_2_ into its biological inactive metabolite [62]. Lu et al. showed that increased miR-21 expression promoted cholangiocarcinogenesis and tumour progression in vitro and in vivo by inhibiting the expression of 15-PDGH and thereby antagonizing the pro-inflammatory and tumour effect of PGE_2_ [67].

miR-21 has been found to induce tumour progression in CCA by inducing epithelial-mesenchymal transition (EMT); a process in which polarised epithelial cells undergo a sequence of biochemical changes that cause them to lose their epithelial properties and develop invasive mesenchymal phenotype. Cancers cells undergo EMT to spread to secondary sites away from the primary site. Li et al. showed that overexpression of miR-21 induced EMT in human CCA cell lines as demonstrated by the loss of expression of epithelial biomarker E-cadherin and the acquisition of the mesenchymal biomarkers N-cadherin and vimentin in vitro and in vivo [68]. A different paper by Liu et al. showed that miR-21, in combination with the transcription factor Krüppel-like factor 4 (KLF4), increased the EMT process in CCA via AKT/ERK1/2 pathway [69]. More recently, miR-21 was shown to modulate CCA drug response to Heat Shock protein inhibitors in cell lines, mouse models and patient-derived organoids, underlining the potential of ncRNAs to inform therapy [70]. These studies illustrate the oncogenic role of miR-21 in inducing carcinogenesis, migration, cell proliferation and metastasis in BTC. 

miR-25: the earliest support for the oncogenic role of miR-25 in BTC was a publication by Razumilawa et al. which highlighted that miR-25 promoted resistance to apoptosis in CCA cells via TNF related apoptosis inducing ligand (TRAIL) [71]. TRAIL is a ligand known to bind to its receptors namely, receptor death receptor 4 (DR4). The study indicated that overexpression of miR-25 downregulated the DR4 gene expression, thereby inducing apoptosis resistance in CCA cells. A recent paper by Liu et al. highlighted that the overexpression of miR-25 was linked with poor prognosis in CCA [72]. The study also demonstrated miR-25 promoted migration, cell viability and invasiveness in CCA cells.

miR-26a: the earliest evidence of the oncogenic role of miR-26 came from a study by Zhang et al. which demonstrated that miR-26a was over-expressed in human CCA and lead to an increase in CCA cell proliferation and migration via β-catenin-dependent genes [73]. The study indicated miR-26a targets and reduces the expression of the GSK-3β mRNA leading to activation of β-catenin and several other genes including cyclin D1, peroxisome proliferator-activated receptors (PPARs), c-Myc. This study thus demonstrated that miR-26a induces cholangiocarcinogenesis and proliferation of CCA cells.

miR-1249: changes in miRNA expression in tissues in BTC has been associated with changes in chemosensitivity to various chemotherapy drugs. Carotenuto et al. showed that the inhibition of miR-1249 increased the sensitivity of CCA to cisplatin-gemcitabine chemotherapy drug via the FZD8 gene [74]. Functional high-throughput screening approaches in cells undergoing drug treatment demonstrated that miR-1249 is responsible for the expansion of CD133+ CCA cells that hold features of cancer stem cells and mediate primary and secondary resistance to chemotherapy. This indicates an oncogenic role of miR-1249, an effect in inhibiting the chemosensitivity in BTC and the promise to be a potential target of therapeutic research.

### 3.2. Tumour Suppressor miRNA

miR-34: there is a wide range of tumour suppressor miRNAs identified in BTC and they have different mechanisms of action. Li et al. indicated miR-34 regulates apoptosis and cell cycle arrests in several types of cancers namely breast, prostate, bladder cancer and thus have a tumour suppressor role in reducing tumour growth [83]. A different paper by Jin et al. showed that low levels miR-34a and longer telomere length was associated with poorer prognosis in GBCs [84]. The study also indicated miR-34a regulates telomere length in vivo and in vitro by regulating the *PNTUS* gene [84]. *PNTUS* is a protein known to regulate telomere length and overexpression of *PNTUS* decreases shortening of the telomere. Telomere shortening limits the number of cellular divisions that can occur and thus the *PNTUS* gene is downregulated in GBC. MiR-34 has also been shown to have a role in inhibiting invasiveness and metastasis of human extrahepatic CCA cells by inhibiting EMT [85]. Qiao et al. showed that the activation of miR-34a inhibited EMT in CCA targeting the *SMAD4* gene through TGF-β/Smad pathway [85]. The study also showed activation of miR-34a suppressed the invasiveness and migration of CCA cells.

miR-146b-5p: Cai et al. showed the expression of miR-146b-5p was downregulated in GBC tissue compared to normal tissue [86]. The study also demonstrated that overexpression of miR-146b-5p promoted apoptosis and G1 cell cycle arrest in GBC cells. miR-145-5p was also shown to inhibit the progression of gallbladder cancer by targeting the epidermal growth factor receptor (EGFR). These results indicate a tumour suppressor role of miR-146b-5- in BTC.

miR-125b-5p and miR-145: chemoresistance is a significant cause of therapeutic failure in treating patients with BTC. Yang et al. highlighted a tumour suppressor role of miR-125b-5p in GBC by enhancing GBC cells sensitivity to a chemotherapy drug cisplatin via downregulating *Bcl2* gene [87]. Downregulation of miR-125b-5p expression in vitro resulted in lower cell death when treated with the chemotherapy agent cisplatin while upregulation of miR-125b-5p caused an increase in cisplatin-induced cell death. A recent publication by Zhan et al. indicated that miR-145 had a similar tumour suppressor role in GBC cells by increasing their sensitivity to cisplatin via downregulating the multidrug resistance associated protein 1 (MRP-1) [88]. Lower expression of miR-145 and higher expression of MRP-1 were linked with poorer prognosis in patients treated with chemotherapy. Both studies illustrated the tumour suppressor role of miRNAs in BTC by promoting sensitivity to therapeutic chemotherapy which could be lifesaving in BTC patients [87,88].

## 4. Clinical Implications of miRNA in Biliary Tract Cancer

### 4.1. Circulating miRNA

Numerous studies have shown miRNAs are not only bound to tissues; they can be released into the serum, plasma, bile and other bodily fluids as or circulating miRNAs [54]. A known mechanism by which miRNAs are released into bodily fluids is through extracellular vesicles (EV). EV which include exosomes and micro-vesicles are made of the lipid membrane and are used to facilitate communications between different cells in numerous physiological processes [103]. Heterogeneous nuclear ribonucleoproteins regulate the loading of miRNAs into EVs via recognising the EXOmotif of miRNAs [104]. The EVs protect miRNAs from being degraded by extracellular RNAses and induce stabilisation of miRNAs. Recent evidence has demonstrated that miRNA can also be released without EVs into the extracellular environment as freely circulating miRNAs and argonaut RISC-bound miRNA [105]. The properties of extracellular miRNAs have raised the interest of the potential of utilising circulating miRNAs as biomarkers in the clinical management of BTC patients [106].

### 4.2. Circulating miRNA as Biomarkers in Biliary Tract Cancer

Late diagnosis of BTC is one of the major contributors to its poor clinical prognosis and low survival rate in patients. Late diagnosis of BTC is due to several factors; firstly, unspecific symptoms because most BTC patients present with similar symptoms as benign biliary disease (BBD), e.g., primary sclerosing cholangitis (PSC), choledocholithiasis, etc. Secondly, the current methods used in diagnosing BTC diagnosis such as imaging, laparoscopy, cholangioscopy are mainly invasive and have low sensitivity in detecting BTC at its early stage due to the anatomical location of these tumours [107]; thirdly, the current biomarkers of BTC used in clinical practice, CA19-9 and carcinoembryonic antigen (CEA) have low sensitivity and specificity in detecting BTC [108,109].

Early non-invasive biomarkers have the potential to improve prognosis and increase overall survival in BTC patients (Table 3). Circulating miRNAs show potential to be early non-invasive diagnostic biomarkers because they have great stability in different bodily fluids and are not easily degraded by RNAses [110]. miRNA expression in BTC patients is significantly different from healthy individuals or non-malignant counterparts. This suggests that a change in circulating miRNA expression in bodily fluids has the potential to differentiate between both groups and can be used to develop diagnostic assays.

Li et al. studied the circulating miRNA profile in GBC patients; expression of oncogenic miR-21, miR-187 and miR-202 was upregulated and the expression of tumour suppressor miR-143 and miR-335 were downregulated in the serum of GBC patients compared to healthy volunteers [111]. This study demonstrated the potential of using miRNA expression in the serum to diagnose cancer patients via profiling the miRNA expression in the serum of patients at risk. As previously said, a major contributor to late diagnosis in BTC patients is the challenging differential diagnosis with other diseases such as PSC. A study by Bernuzzi et al. illustrated that two circulating miRNAs (miR-483–5p and miR-222) in the serum differentiated CCA patients from PSC patients with an area under the curve AUC of 0.77 [112].

There are however several limits in miRNA serum studies; the miRNAs present in the serum may not be specific to CCA and could be induced by other diseases such as kidney disease, liver disease, heart disease, sepsis, immunological disease, other types of cancer, etc. and that makes it a challenging diagnostic test to use in a clinical setting. Investigating the circulating miRNAs in the bile may provide a pathway to increase the accuracy, sensitivity and specificity of the biomarker because the circulating miRNA expression in bile is less likely to be induced by other diseases. Shigehera et al. examined the 10 circulating miRNAs in bile and demonstrated that a composition of two miRNAs (miR-9, miR-145) differentiated BTC patients from patients with choledocholithiasis [113]. Utilising Multivariate Organization of Combinatorial Alterations (MOCA), Li et al. investigated the miRNA expression of the bile and showed a panel of 5 circulating miRNAs (miR484, miR-19, miR-16, miR-1274b and miR-486–3p) differentiated CCA patients from PSC patients with a sensitivity of 67% and specificity of 96% [114].

There are challenges to investigating miRNA expression in bile samples. Firstly, the bile extraction is a very invasive procedure that requires a medical procedure. This causes a technical feasibility problem because bile is not collected from healthy patients (with no malignant lesion) and this leads to a shortage of biological samples of healthy patients available to researchers. Most researchers rely on the miRNA expression profile in already collected bile samples from BBD patients to develop diagnostic assays. A method to solve this problem is to measure the circulating miRNA expression in other bodily fluids that are more easily accessible, e.g., urine samples. Circulating miRNAs are known to be present in urine samples due to high stability and could potentially be a less invasive diagnostic test. A paper by Silakit et al. showed that the expression of miR-192 was greater in the urine samples of *Opisthorchis viverrini*-infected patients compared to healthy volunteers, having a sensitivity of 75%, specificity of 71.4% and AUC of 0.766 [115]. While these results are not good enough to be used in clinical practice as a diagnostic biomarker, it is better than CA19-9 which has an AUC of 0.723 and it shows the potential of examining miRNA expression in urine samples in other diseases [109]. The accuracy of the diagnostic test can be improved by measuring the expression of multiple miRNAs thereby improving the sensitivity and specificity. For example, in the same study, when miR-21was combined with miR-192, it increased the sensitivity to 79.2%, specificity to 76.2 and AUC to 0.812.

miR-21 plays a huge role in BTC and thought to be potentially one of the best diagnostic biomarkers of BTC. Kishimoto et al. explained that circulating miR-21 could differentiate between BTC and BBD patients in serum with a sensitivity of 72.3%, specificity of 91.3% and AUC of 0.83 [116]. However, miR-21 is expressed in multiple types tumours, e.g., gastric cancer, liver cancer, breast cancer, prostate cancer [117,118,119,120] and therefore lacks specificity to be used alone in clinically diagnosing BTC. More specific miRNAs or miR-21 in combination with other miRNAs may provide the best pathway to an accurate clinical diagnostic tool.

There are many challenges in clinically utilising miRNA expression as a diagnostic biomarker. Firstly, there are known inconsistencies in miRNA expression in tissues compared to the circulating miRNAs in bodily fluids. Studies have demonstrated specific miRNAs are expressed in tissues but not detected in bodily fluids and vice versa. These problems are likely due to variations in the different studies, e.g., specimen collection, data reporting, experimental techniques, etc. The specimen collection and experimental techniques have to be standardised to discover accurate diagnostic biomarkers for clinical practice. The most accurate way to do this is by counting the molecules of miRNA in a given bodily fluid. Recent advancement in technology has provided quantitative PCR which provides a pathway to solve this problem because it can accurately quantitate the miRNA molecules in bodily fluids and a threshold can be identified for validation. Another challenge is the discrepancies in the results and data across the literature; for example, some papers indicated certain miRNAs are expressed in the bodily fluid of patients and other papers did not. A possible cause of this is the small cohort of patients in various studies. Studies with large cohorts of patients involving all age groups, sexes, races will be required to further investigate the diagnostic potential of circulating miRNAs.

### 4.3. miRNA-Based Therapeutics

The role of miRNA in cancer and its propensity to target numerous genes and signalling pathways in carcinogenesis has made miRNA a potentially attractive therapeutic target for researchers. The strategies for the development of miRNA-based therapeutics can mainly be divided into two [125]; firstly, anti-miRNA-based therapeutics or miRNA inhibitors that target the oncogenic miRNAs. Secondly, a synthetically derived miRNA (miRNA mimics) that mimics or replenishes the tumour suppressive miRNAs. Another therapeutic agent that targets miRNA is enoxacin. It is an antibacterial compound that has been shown to modulate RNAi pathway via the siRNA mediated degradation of mRNA and stimulation of endogenous miRNA biogenesis [126]. A subsequent study identified anti-tumour properties of enoxacin that were attributed to its effect on RNAi pathways [127].

There are several vital procedures in the advancement of miRNA-based therapeutics and each step has major challenges. Firstly, identifying the right miRNA candidates and their targets; this is an important step in producing an effective therapy with little or no off-target effects and it requires systematic evaluation of miRNA expression in both tissue and bodily fluids. A major challenge in identifying miRNAs therapeutics is that most miRNAs and their targets are not cancer-specific; they can be targeted by other conditions, e.g., hypoxia, heart disease, inflammation, etc. so there is a huge potential of having off-targets and toxic side effects [10,56,128]. The recent advancement of genomics and new sequencing approach has provided a wide range of systemic database, e.g., the public repository of cancer integrative “omics” data (TCGA), that has the potential to improve the process of identifying the best possible miRNA targets [129]. However, there still remains the need for a more comprehensive and encompassing repository for miRNA that covers their pleiotropic nature and their context-specific targets in different cancers to obtain a wholistic view of their clinical relevance. In addition, the functional activity of miRNAs may depend on the tissue-specific stoichiometry of mRNA transcripts, underlining the need to verify the expression and function of each miRNA in the specific tumor type before moving it ahead as a therapeutic candidate. A novel technique of miRNA and crosslinking immunoprecipitation (miR-CLIP) shows potential for more precise identification of important miRNA pathways by capturing the miRNA targetome [130].

A major challenge in clinically utilising miRNA-based therapeutics is that they can be degraded by nucleases such as RNAses in the bodily fluids. A method to solve this problem is to induce chemical modifications to miRNA-based therapeutics to promote miRNA stability in the presence of nucleases. Chemical modifications that have been made to miRNA include methylation to change oligonucleotide chemistry, the incorporation of locked nucleic acids and the insertion of phosphorothioate-like groups. In a clinical study, a 2′O-methoxyethyl modification was introduced in anti-MIR against miR-122, showing better resistance to nucleases than the unmodified anti-MIR [131]. While these modifications improve the miRNA resistance to nucleases, it has been shown to more likely lead to a reduction in the ability of miRNA to silence its target genes and also increases the off-target side effects. A different strategy is the development of exosomes or micro-vesicles like vehicles or nanoparticles to transport miRNA-based therapeutics into bodily fluids. This strategy has the potential to protect the miRNA from nucleases in bodily fluids and limit the off-target effects. An ideal miRNA vehicle will be non-immunogenic, durable and have limited off-target effects. There has been loads of research into numerous potential vehicles for miRNA mimics and they include viral vectors, neutral liposome 1,2­dioleoyl­sn­glycero­3­phosphatidylcholine DOPC, EnGeneIC delivery vehicle nanocells, synthetic polyethylenimine, chitosan, dendrimers, neutral lipid emulsions, cyclodextrin, poly(lactide­co­glycolide) particles, N­acetyl­D­galactosamine, chitosan [125].

There have been several pre-clinical, phase I and II trials investigating the safety of different miRNA-based therapeutics in the treatment of different types of cancer and other diseases including hepatitis C infection, cardiovascular disease, atherosclerosis, etc. In a preclinical trial, a miR-200c mimic in a DOPC carrier was shown to significantly improve the overall survival of an orthotopic mouse model of lung cancer compared to control [132]. miR-200c mimic was demonstrated to inhibit *ZEB1* gene and genes encoding the oxidative stress response leading to apoptosis. A different preclinical trial examined the effect of a chemically modified antimir antisense oligonucleotide (ASO) that targets the oncogenic miR-10b in an orthotopic model of breast cancer [133]. Although the results from the trial showed a reduction in metastasis of the breast cancer cells, it did not show a reduction of primary tumour growth. This suggests a miR-10b inhibitor should be used alongside other treatments such as chemotherapy or surgery.

MRX34 is a miR-34a mimic and it was encapsulated in lipid carrier known as NOV40. The benefit of using NOV40 particles as a miRNA vehicle is NOV40 is positively charged in a tumour microenvironment and that allows it to bind with tumour cells [132]. Studies that investigated the effect MRX34 nanoparticles in mice showed increased expression of miR-34 and a significant reduction in tumour growth [134,135,136,137]. The positive results of the trial demonstrated the potential of MRX34 in treating cancer in humans and subsequently led a multicentre dose escalation/expansion phase 1 trial that tested efficacy and safety of MRX34 in patients with conditions including small cell lung cancer, primary liver cancer, melanoma, lymphoma, renal cell carcinoma, multiple myeloma, non-small cell lung cancer [138]. In the initial stage of the phase 1 trial, MRX34 was administered to patients via intravenous infusion in a 2 times per week in 4 weeks cycles. In the final stage of the trial, MRX34 was administered along with dexamethasone daily for 5 days in a 3 weeks cycle. An aggregate of 85 patients with hepatocellular carcinoma HCC, melanoma, renal cell carcinoma, lung cancer, gastrointestinal stromal tumour and neuroendocrine cancer were included in the study. At the end of the trial, 3 patients had a prolonged confirmed partial response and 16 patients had stable disease. Pharmacodynamic analysis of white blood cells showed that miR-34 targeted mRNAs in white blood such as forkhead box P1 (FOXP1) and BCL2.

However, MPX34 had an adverse safety profile; common side effects noted in the 85 patients enrolled in the study included fever, chills, fatigue, back/neck pain, nausea and dyspnoea. The study was later terminated as a result of significant immune-mediated adverse effects that resulted in the death of 4 patients. However, the dose-dependent regulation of target genes showed the potential of miRNA-based therapeutics (Clinical trial information: NCT01829971). As previously discussed, studies have indicated miR-34 has a tumour suppressor role in BTC and therefore there is a potential for miR-34 mimic in treating BTC patients [84,85,90]. However, more research has to be done with a special focus on understanding the cause and prevent or treat the immunogenic side effects are required to truly improve this research field.

There are potential strategies made possible by the advancement of technology that can address the toxic side effects of RNA-based therapeutics. RNA therapies available today are mainly divided into three groups [139]; RNA therapeutics that target and silence DNA or RNA, e.g., through RNA interference (siRNA, miRNA), those that target proteins, e.g., RNA aptamer, and finally those that encode proteins. Some researchers are looking to combine all three groups i.e., RNA therapeutics that silence or degrade unfavourable genes, e.g., Using RNAi and replace the mRNA by encoding the genes. The past experience with microRNA-based therapeutics has shown toxicity as a main limiting factor [140]. However, recent advancements in the design and delivery of RNA therapeutics (Table 4) has opened up new avenues for optimization of this strategy. Indeed, many RNA therapeutics have now been approved [141] and trials are ongoing for several diseases [142].

## 5. Future Perspectives

A major challenge in utilising circulating miRNA as biomarkers or targeted for therapeutics is the heterogeneity of miRNA expression. Circulating miRNA expression can be induced by other physiological processes such as hypoxia and inflammation, and that makes it harder to ascertain that variation in miRNA expression are specific for BTC. Advancement in genomics and new sequencing approach has provided a wide range of systemic database to improve the process of identifying the right miRNA targets and pathways. However, these databases have to be continuously improved and updated with new information as they become available to make better headway in accurately identifying miRNA targets. miR-CLIP, a novel crosslinking and immunoprecipitation technique, can potentially improve this process of identifying accurate miRNA changes in expression in BTC.

Another challenge is the discrepancies in data from the studies, which make it difficult to assert suitable miRNA targets for biomarkers and therapeutics. This is most likely as a result of too little number of patients in the studies and lack of standardisation of the available data. Using large cohorts of patients in studies could be helpful. This requires international cooperation from researchers as it remains difficult to find the adequate patient populace in any health care setting or a single country. International consortia can play a significant role in bringing together researchers and patients to provide the largest possible studies for faster and more accurate results. The advancement in modern technology has produced digital quantitative PCR which provides a pathway to count miRNA molecules in biofluids and standardised the data for validation.

An approach that has not yet been extensively looked into is the clinical implications of the immunogenic effects of ncRNA. It is known miRNA plays a role in inflammation in response to physiological processes such as cancer [143]. Changes in miRNA expression that leads to poor immunological responses in response to BTC may provide a pathway for early predictive, prognostic and diagnostic biomarkers of BTC. With early diagnosis, the appropriate treatment can be provided to patients to improve clinical prognosis and overall survival in patients.

## 6. Conclusions

Biliary tract cancer generally has a very poor clinical prognosis as most patients survive less than a year in its advanced stages. This is because of the limited understanding of its pathogenesis of BTC, late diagnosis, and limited therapeutics in the treatment and management of BTC. Recent studies in BTC tissues and cells have demonstrated ncRNA have important roles in the pathogenesis of BTC. Studies have also shown miRNA can be expressed as circulating miRNA in bodily fluids. This led to research into the potential of tissue-bound and circulating miRNA as biomarkers and therapeutics for BTC. The results from recent studies have shown miRNA could potentially be employed as clinical diagnostic biomarkers and targeted for therapeutics in the diagnosis and treatment of BTC respectively but there are many challenges.

In summary, research into the clinical implications of miRNA in BTC is still in its early phase and extensive research is required for miRNA-based biomarkers and therapeutics to be used in clinical practice.

## Figures and Tables

**Table 1:** Oncogenic miRNAs and their role in biliary tract cancer.

miRNA	Expression	Tumour Type	Targets	Functions	Source	References
miR-20a	Up	GBC	*Smad7*	Invasion, metastasis, prognosis	Tissue, cell	[75]
miR21	Up	CCA, GBC	*PIPN14, PTEN, PI3K, TIMP3, KLF4, 15-PGDH, AKT, ERK*	Proliferation, tumour growth, invasion, migration, EMT	Tissue, cell	[65,66,67,69,76]
miR-25	Up	CCA	*DR4*	Anti-apoptotic	Tissue, cell	[71]
miR-26a	Up	CCA	*GSK-3*	Tumour growth	Tissue, cell	[73]
miR-31	Up	iCCA	*RASA1*	Proliferation, anti-apoptotic	Tissue, cell	[77]
miR-24	Up	iCCA, eCCA	*MEN1*	Proliferation, migration, angiogenesis	Tissue, cell	[78]
miR-141	Up	CCA	*CLOCK*	Proliferation	Tissue, cell	[64]
miR-182	Up	GBC	*CADM1*	Migration, invasion, metastasis	Tissue, cell	[79]
miR-221	Up	eCCA	*PTEN*	Invasion, migration, EMT	Tissue, cell	[80]
miR-421	Up	CCA	*FXR*	Proliferation, migration	Tissue, cell	[81]
miR-1249	Up	CCA	*FZD8*	Chemoresistance	Tissue, cell	[74]
Let-7a	Up	CCA	*NF2*	Survival	Tissue, cell	[82]

Abbreviations: *Smad7*, mothers against decapentaplegic homolog 7; *PTPN14*, protein tyrosine phosphatase, non-receptor type 14; PTEN, phosphatase and tensin homolog; PI3K, phosphoinositide-3-kinase; *TIMP3*, TIMP metallopeptidase inhibitor 3; *KLF4*, Kruppel-like factor 4; *15PGDH*, 15-hydroxyprostaglandin dehydrogenase; *Akt*, AKT serine/threonine kinase; *ERK*, extracellular signal-regulated kinase; *DR4*, death receptor 4; *GSK-3β*, glycogen synthase kinase 3 beta; *RASA1*, RAS P21 Protein Activator 1; *MEN*, Menin; *NF2*, neurofibromin 2; *CLOCK*, circadian locomotor output cycles kaput protein; *CADM1*, cell adhesion molecule 1; *FXR*, farnesoid X receptor; *FZD8,* Frizzled 8; *NF2*, neurofibromin 2; GBC, gallbladder cancer; CCA, cholangiocarcinoma; iCCA, intrahepatic cholangiocarcinoma; eCCA, extrahepatic cholangiocarcinoma; EMT, epithelial-mesenchymal transition.

**Table 2:** Tumour suppressor miRNA and their role in biliary tract cancer.

miRNA	Expression	Tumour Type	Targets	Functions	Source	References
miR-29b	Down	CCA	*Mc11*	Anti-apoptotic	Cell	[89]
miR-34a	Down	eCCA, GBC	*Per-1, SMAD4, PNTUS*	Proliferation, invasion, migration, EMT, lymph node metastasis	Tissue, cell	[84,85,90]
miR-101	Down	CCA, GBC	*COX-2, VEGF*	Angiogenesis, invasion, survival	Tissue, cell	[91]
miR-122	Down	GBC	*PKM2*	Lymph node metastasis, pTNM stage	Tissue, blood	[92]
miR-125b-5p	Down	GBC	*Bcl2*	Chemoresistance	Tissue, cell	[87]
miR-146b-5p	Down	GBC	*EGFR*	Proliferation, apoptosis, tumour size	Tissue, cell	[86]
miR-144	Down	CCA	*LIS1*	Proliferation, invasion, migration	Tissue, cell	[93]
miR-145	Down	GBC	*MRP-1, AXL*	Proliferation, apoptosis, poor survival, chemoresistance	Tissue, cell	[90,94]
miR-200	Down	CCA, iCCA	*SUZ12, ROCK2, NCAM1*	Invasion, migration, EMT, drug resistance	Tissue, cell	[95]
miR-218-5p	Down	GBC	*Bmi1*	Proliferation, migration, chemoresistance	Tissue, cell	[96]
miR-370	Down	CCA	*MAP3K8, WNT10B*	Proliferation	Tissue, cell	[97,98]
miR-373	Down	pCCA	*MBD2*	Proliferation	Tissue, cell	[99]
miR-410	Down	CCA	*XIAP*	Proliferation	Tissue, cell	[100]
miR-494	Down	CCA	*CDK6, PLK1, CDC20, TOP2A, CCNB1, CDC2, PTTG1*	Proliferation	Tissue, cell	[101,102]

Abbreviation: *Mcl-1*, myeloid cell leukemia sequence 1; *Per-1*, period circadian regulator 1; *SMAD4*, SMAD family member 4; *PNUTS*, phosphatase nuclear targeting subunit; *COX-2*, cyclooxygenase-2; *DNMT-1*, DNA methyltransferase 1; *VEGF*, vascular endothelial growth factor; *PKM2*, pyruvate kinase isozymes M2; *Bcl-2*, B cell CLL/lymphoma 2; *EGFR*, epidermal growth factor receptor; *LIS1*, lissencephaly-1 homolog; *MRP-1*, multidrug resistance-associated protein 1; *AXL*, AXL receptor tyrosine kinase; *SUZ12*, SUZ12 polycomb repressive complex 2 subunit; *ROCK2*, Rho associated coiled-coil containing protein kinase 2; *NCAM1*, neural cell adhesion molecule 1; *Bmi1*, B-cell-specific moloney murine leukemia virus integration site 1; *MAP3K8*, mitogen-activated protein kinase kinase kinase 8; *WNT10B*, Wnt family member 10B; *MBD2*, methyl-CpG binding domain protein 2; *XIAP*, X-linked inhibitor of apoptosis; *CDK6*, cyclin dependent kinase 6; PLK1, polo like kinase; *CDC20*, cell division cycle 20; *TOP2A*, DNA topoisomerase II alpha; *CCNB1*, cyclin B1; *CDC2*, cell division control protein 2 homolog;; *pTNM*, pathological tumour/node/metastasis; pCCA, perihilar cholangiocarcinoma.

**Table 3:** Circulating miRNAs as potential biomarkers.

miRNA	Source	Clinical Setting	Expression	Impact	References
miR-21	Plasma	BTC vs HV	Up	84% SV 98% SP 0.93 AUC	[116]
miR-21	Plasma	BTC vs BBD	Up	71.2% SV 82.6% SP 0.83 AUC	[116]
miR-150	Plasma	iCCA vs HV	Up	80.6% SV 58.1% SP 0.764 AUC	[121]
miR-192	Plasma	iCCA vs HV	Up	74% SV 72% SP 0.803 AUC	[122]
miR-21 + CA19-19	Plasma	BTC vs BBD	Up	79.8% SV 91.3% SP	[116]
miR-1281, miR-26a, miR-122	Serum	CCA vs PSC	Up	32–68% SV 88–93% SP	[123]
miR-412, miR-680, miR-1537, miR-3189	Bile	CCA vs PSC	Down	50–67% SV 89–92% SP	[123]
miR-106a	Serum	CCA vs BBD	Down	56.3% SV 100% SP 0.79 AUC	[124]
miR-192	Urine	OPV vs HV	Up	75.0% SV 71.4% SP 0.766 AUC	[115]
miR-192 + miR-21	Urine	OPV vs HV	Up	79.2% SV 76.2% SP 0.812 AUC	[115]
miR-192 + miR-21	Urine	OP-CCA vs HV	Up	81.2% SV 71.4% SP 0.849 AUC	[115]

Abbreviations: BTC, biliary tract cancer; BBD, benign biliary disease; HV, healthy volunteers; OPV, *Opisthorchis viverrini*-infected patients; CCA, cholangiocarcinoma; Op-CCA, opisthorchis’s-associated cholangiocarcinoma; PSC, primary sclerosing cholangitis; SP, specificity; SV, sensitivity; AUC, Area under the curve.

**Table 4:** RNA therapeutics in clinical trials. Phase III trials are presented in all diseases, along with all phase trials in cancer.

Candidate	Type of RNA Therapeutics	Biological Target	Disease	Clinical Stage	Clinical Trial Number
QR-110	ASO	Centrosomal protein 290	Leber’s congenital amaurosis	Phase II/III	NCT03913143
Inclisiran	siRNA	Proprotein convertasesubtilisin/kexin type 9	Atherosclerotic cardiovascular disease Hypercholesterolemia Elevated LDL	Phase III Phase III Phase III	NCT03399370 NCT03851705 NCT03814187
Lumasiran	siRNA	Hydroxyacid oxidase 1	Primary hyperoxaluria type 1	Phase III	NCT04152200
Fitusiran	siRNA	Antithrombin	Hemophilia	Phase III	NCT03549871
Patisaran	siRNA	Transthyretin	Transthyretin Amyloid	Phase III	NCT03997383
Vutrisiran	siRNA	Transthyretin	Transthyretin Amyloid	Phase III	NCT04153149
JNJ-3989	siRNA	Hepatitis B surface antigen	Chronic Hepatitis B infection	Phase II	NCT04439539
STP705	siRNA	TFGb, COX2	Cutaneous Squamous Cell Carcinoma in Situ	Phase I/II	NCT04293679
SAR339375	Anti-miR	miR-21	Alport’s syndrome	Phase II	NCT02855268
Olaptesed	Aptamer	Chemokine (CXC motif) ligand 12	Glioblastoma	Phase I/II	NCT04121455
CV81012	ncRNA	Activation of TLR7/8/RIG-1	Solid Tumours	Phase I	NCT03291002

Abbreviations: ASO: antisense oligonucleotide. siRNA: small interfering RNA. ncRNA: non-coding RNA.
